# Developmental outcome of very low birth weight infants in a developing country

**DOI:** 10.1186/1471-2431-12-11

**Published:** 2012-02-01

**Authors:** Daynia E Ballot, Joanne Potterton, Tobias Chirwa, Nicole Hilburn, Peter A Cooper

**Affiliations:** 1 Department of Paediatrics and Child Health, University of the Witwatersrand, PO Wits, 2050, South Africa; 2 Department of Physiotherapy, University of the Witwatersrand, PO wits, 2050, South Africa; 3 Epidemiology and Biostatistics Division, School of Public Health, University of the Witwatersrand, PO Wits, 2050, South Africa

## Abstract

**Background:**

Advances in neonatal care allow survival of extremely premature infants, who are at risk of handicap. Neurodevelopmental follow up of these infants is an essential part of ongoing evaluation of neonatal care. The neonatal care in resource limited developing countries is very different to that in first world settings. Follow up data from developing countries is essential; it is not appropriate to extrapolate data from units in developed countries. This study provides follow up data on a population of very low birth weight (VLBW) infants in Johannesburg, South Africa.

**Methods:**

The study sample included all VLBW infants born between 01/06/2006 and 28/02/2007 and discharged from the neonatal unit at Charlotte Maxeke Johannesburg Academic Hospital (CMJAH). Bayley Scales of Infant and Toddler Development Version 111 (BSID) 111 were done to assess development. Regression analysis was done to determine factors associated with poor outcome.

**Results:**

178 infants were discharged, 26 were not available for follow up, 9 of the remaining 152 (5.9%) died before an assessment was done; 106 of the remaining 143 (74.1%) had a BSID 111 assessment. These 106 patients form the study sample; mean birth weight and mean gestational age was 1182 grams (SD: 197.78) and 30.81 weeks (SD: 2.67) respectively. The BSID (111) was done at a median age of 16.48 months. The mean cognitive subscale was 88.6 (95% CI: 85.69 - 91.59), 9 (8.5%) were < 70, mean language subscale was 87.71 (95% CI: 84.85 - 90.56), 10 (9.4%) < 70, and mean motor subscale was 90.05 (95% CI: 87.0 - 93.11), 8 (7.6%) < 70. Approximately one third of infants were identified as being at risk (score between 70 and 85) on each subscale. Cerebral palsy was diagnosed in 4 (3.7%) of babies. Factors associated with poor outcome included cystic periventricular leukomalacia (PVL), resuscitation at birth, maternal parity, prolonged hospitalisation and duration of supplemental oxygen. PVL was associated with poor outcome on all three subscales. Birth weight and gestational age were not predictive of neurodevelopmental outcome.

**Conclusion:**

Although the neurodevelopmental outcome of this group of VLBW infants was within the normal range, with a low incidence of cerebral palsy, these results may reflect the low survival of babies with a birth weight below 900 grams. In addition, mean subscale scores were low and one third of the babies were identified as "at risk", indicating that this group of babies warrants long-term follow up into school going age.

## Background

Advances in neonatal care allow survival of extremely preterm infants, who are prone to a range of long term complications in comparison to their term counterparts [1-4]. These problems range from severe handicap such as cerebral palsy, cognitive impairment, blindness and hearing loss to impairment of short term memory, strabismus, language delays, learning difficulties and behavioural disorders [2,5,6]. Individual children often have multiple disabilities [7] and these handicaps persist into school going age and beyond [8,9]. There is concern that improved rates of survival of very low birth weight (VLBW), and particularly extremely low birthweight (ELBW) infants, may be associated with increased rates of neurodevelopmental handicap [10], although some report improved survival without increased handicap [11].

Ongoing long term neurodevelopmental follow up of preterm infants and current outcome data [12], with analysis in changes of outcomes over time and between different regions, are essential and must form part of the evaluation and safety monitoring of new interventions and technological advances in neonatal care [13]. Advances in neonatal care are readily adopted by developing countries, which often have poorly resourced health services. The concern about long term outcome and safety monitoring of neonatal interventions in this setting is equally applicable. Rates of handicap may be far higher than those reported from the First World. A study from Bangladesh reported that only 32% of infants born at < 33 weeks were developmentally normal at 12 months of age [14]. Outcome data from VLBW infants managed in a first world setting cannot simply be extrapolated to an under resourced setting, except possibly as a goal to work towards. It is essential to have current outcome data from the infants managed in under resourced settings in order to properly manage neonatal care in that situation. There is a paucity of current published data on long term outcome of VLBW from developing countries, including South Africa. Cooper and Sandler conducted such a study in the early 1990s [15]. They found that there was a high incidence of post discharge mortality, but that rates of handicap were similar to those in developed countries. There have been major changes in South Africa since then, both socio-political and health related, including implementing free health care for mothers and children less than 6 years of age, the introduction of surfactant therapy and nasal continuous positive airway pressure (NCPAP), standard use of antenatal steroids and establishing kangaroo mother care (KMC).

The aim of this study was to determine developmental outcome in a cohort of VLBW infants at Charlotte Maxeke Johannesburg Academic Hospital (CMJAH), as measured by the Bayley Scales of Infant and Toddler Development (third edition) (BSID 111) [16] and to determine factors associated with poor outcome.

## Methods

All VLBW infants born between 2006/06/01 and 2007/02/28 treated at the CMJAH neonatal unit who survived to discharge were invited to participate in the follow up study. The following infants were excluded: babies who were transferred to step-down facilities before discharge, those whose parents were relocating to other areas and would not be available for follow up at CMJAH, babies who were put up for adoption and those whose parents refused consent. The study was approved by the Ethics Committee of the University of the Witwatersrand for Research on Human Subjects. Written informed consent was obtained from each baby's parents before being entered into the study.

### Patient characteristics

All babies were admitted into the neonatal unit of CMJAH and care was according to standard protocols. Resuscitation at birth refers to the need for bag mask ventilation with or without chest compressions. Gestational age was assessed by attending staff using a combination of maternal history and Ballard scoring. The need for nasal continuous positive airways pressure (NCPAP), surfactant therapy and intubation with mechanical ventilation was at the discretion of the attending physician. Intermittent positive pressure ventilation (IPPV) was used. High frequency oscillation and jet ventilation were not available in the unit at the time. Ventilatory support, including NCPAP, with or without surfactant therapy, was only given to babies with a birth weight above 900 grams. Babies below 900 grams were given all other care, but not ventilation. This cutoff for ventilation is determined by limited health resources and is well established in neonatal practice in South Africa. All infants were resuscitated at birth if needed, regardless of birth weight.

Patent ductus arteriousus (PDA) was confirmed on echocardiogram when suspected clinically. Intraventricular haemorrhage (IVH), graded according to Papile [17], and cystic periventricular leukomalacia (PVL) [18] were diagnosed on serial cranial ultrasound examinations done by a paediatric neurologist. Magnetic resonance imaging was not available at the time. Necrotising enterocolitis (NEC) was graded according to modified Bell's staging [19]. Early sepsis was defined as a baby with clinical signs of sepsis and a positive blood culture presenting within 72 hours of birth, late onset sepsis after 72 hours. The continuous kangaroo mother care (KMC) unit was not open at the time of the study, so KMC was done intermittently at the bedside once the baby was stable and off all intravenous therapy. Babies receiving oxygen via nasal cannula could receive KMC, but not those on NCPAP or ventilation. It was noted in the unit that intravenous lines and NCPAP easily pulled out in infants undergoing KMC, so nursing staff preferred to wait until the baby was on full enteral feeds and off NCPAP prior to initiating KMC.

The hospital records of each patient were reviewed and maternal obstetric information, details of the mode and place of delivery, labour room information and details of the infant's hospital stay were all recorded.

Infants were seen by a paediatrician at a dedicated follow up clinic at 3 monthly intervals until a corrected age of 15 to 18 months was attained. For purposes of the study, the chronological age of the baby was corrected for the degree of prematurity, using a gestational age of 40 weeks as term. Children with health and/or developmental problems were followed up for longer and referred to appropriate specialist clinics as needed. Parents/caregivers were provided with a small transport allowance at each follow up visit. Routine follow up included an interval history, systematic examination and growth monitoring at each visit.

A major concern was the anticipated failure of patients to return for follow up after one year. Potential reasons for loss to follow up in this population included parents unable to afford repeated absenteeism from work to attend clinic, children sent to rural areas to live with grandparents, financial constraints and the perception that children are well and do not need follow up. For this reason, two developmental assessments were done - one between the ages of 8 and 12 months and another 6 months later.

The developmental assessment was done using the BSID 111, by one of two neurodevelopmentally trained physiotherapists (NH/JP) who were blinded to the details of the patient's birth and hospital admission. Inter observer standardization was done between the two testers; a 98% agreement was achieved between the results.

Defaulters were contacted both telephonically and by mail to encourage them to return for follow up. The reasons for defaulting and the child's general condition were obtained if possible. If a defaulter returned to the follow up clinic, the developmental assessment was done at that visit.

### Statistical analysis

Descriptive statistics and analysis to determine factors associated with developmental outcomes were performed using STATA version 10 (StataCorp 2007. *Stata Statistical Software: Release 10*. College Station, TX: StataCorpLP). Frequency tabulations and percentages for categorical data such as gender, mode of delivery and HIV status were produced to describe patient characteristics. For continuous data, summary measures such as mean and standard deviation (SD) or 95% confidence intervals for normally distributed data or median and inter-quartile range (IQR) for non-normally distributed data were presented. Cross-tabulations of patient characteristics with each of the abnormal Bayley scales are also presented. The BSID 111 does not have a single composite outcome. There are 3 separate subscales - motor, cognitive and language. An abnormal outcome on each subscale is a score < 70, and those at risk are considered to have a score of between 70 and 85. In line with standard reporting an abnormal score for regression analysis would be considered as a score of ≤ 85. Associations between patient and clinical characteristics with each abnormal outcome were investigated using the Chi-squared test at 5% level of significance.

A number of patients defaulted on follow up visits after one year; some patients only attended after long periods of defaulting, so not all patients had two BSID 111 scales done and Bayley assessments were done in different ages in different patients. The age of assessment influences the results of the Bayley assessments, which tend to decrease with time [20]. In patients where more than one assessment was done, the latest Bayley assessment score was used as the outcome.

Both univariate and multiple regression analyses were conducted on the following potential risk factors to establish associations with poor outcome: obstetric risk factors, infant demographics, labour room risk factors, neonatal morbidity, therapeutic interventions and duration of hospitalization. Regression analysis was done as follows: Logistic regression was performed on the various risk factors for each subscale considering a score of ≤ 85 to be an abnormal outcome. Linear regression was done for the various risk factors for each subscale, considering the actual score on a continuous scale. To investigate factors associated with each BSID 111 subscale, univariate regression models were fitted. Any factor which was univariately associated with each outcome at a conservative 20% significance level, using a t test, was considered further in the multiple regression model building. The final adjusted model of factors associated with each BSID 111 scale was obtained using 5% significance level cut-off.

## Results

### Final sample

Three hundred and fourteen VLBW babies were admitted to CMJAH during the study period - 92 (29%) died before discharge, 44 (14%) were transferred to regional step-down facilities and 178 (56.6%) were discharged home. Details by birth weight category are shown in Table 1. The 178 babies discharged home from CMJAH were eligible for enrolment in the study; of these, 5 babies were put up for adoption, families of 17 babies relocated, 1 mother could not get time off work to attend follow-up and consent was not obtained in 3 patients. Thus, 152 babies were available to participate in the follow up study. Nine babies died (5.9%) before the first BSID 111 assessment could be performed. This left 143 babies who were available for assessment - and at least one BSID 111 assessment was done in 106 babies - giving a follow up rate of 74.6%. These 106 patients constitute the final study sample. Fifty three infants had two Bayley assessments performed - the first at a mean age of 10.83 (SD: 1.06) months and a second at a mean age of 17.74 (SD: 1.79) months.

**Table 1:** Outcome of babies admitted to CMJAH neonatal unit between 01/06/2006 and 28/02/2007 by birth weight category

Birth Weight (grams)	Total	Died	Discharged from CMJAH	Transferred to step down facility	BSID111 assessment done at follow up
**≤ 600**	2	2	0	0	0
**≤ 700**	12	8	4	0	2
**≤ 800**	22	20	2	0	2
**≤ 900**	24	18	5	1	4
**≤ 1000**	35	13	17	5	11
**≤ 1100**	49	9	29	11	19
**≤ 1200**	38	5	29	4	22
**≤ 1300**	37	8	26	3	13
**≤ 1400**	46	3	29	14	16
**≤ 1500**	49	6	37	6	17

### Bayley scales

The latest Bayley assessment was done at a median corrected age of 16.48 months (range 8 to 22 months). The overall results for each subscale are shown in Table 2. Sixteen (15.1%) babies had BSID 111 subscales < 70: 9 (8.4%) had isolated abnormalities (4 cognitive, 3 language, 2 motor); in 4 (3.7%) patients all three subscales were < 70 and in the remaining three patients (2.7%) two of the subscales were < 70 (2 had abnormal motor and language subscales; the remaining 2 had abnormal cognitive and language subscales respectively). The proportion of infants considered at risk (score 70 to 85) was 34.9% for cognitive, 33% for language and 30.2% for motor subscales respectively. The majority of infants had a score above 85 on each subscale (56% for cognitive, 57.6% for language and 62.3% for motor). Four (3.7%) of the babies were diagnosed with cerebral palsy.

**Table 2:** Descriptive results of the latest Bayley assessment subscales

Subscale	Proportion abnormal (score < 70) n (%)	Proportion at risk (70 ≤ score ≤ 85) n (%)	Proportion Normal (score > 85) n (%)	Mean Score	95% Confidence Interval
**Cognitive**	9 (8.5%)	37 (34.9%)	60 (56.6%)	88.64	85.69 - 91.59
**Language**	10 (9.4%)	35 (33.0%)	61 (57.6%)	87.71	84.85 - 90.56
**Motor**	8 (7.6%)	32 (30.2%)	66 (62.3%)	90.05	87.0 - 93.11

### Demographics, labour room, delivery and hospital stay

The mean birth weight and mean gestational age of the study patients was 1182 grams (SD: 197.78) and 30.81 weeks (SD: 2.67) respectively. The median maternal age was 26 years (IQR: 21.5, 32) and the median Apgar score was 8 (IQR: 7, 9). The mean duration of hospital stay was 40.28 days (SD: 15.06) and the median duration of intensive care was 6 days (IQR: 5, 8). Babies were predominantly black African, the majority of the babies, 61 (58%), were female and 49 (46%) were SGA. Details of delivery, labour room and hospital stay are shown in Table 3 including cross-tabulations by each abnormal BSID 111 subscale (≤ 85), using the latest Bayley assessment.

**Table 3:** Overall patient characteristics and their association with outcomes, based on abnormality cut-off (score **≤ 85**) and on each Bayley Scale using latest observation of patients

			Frequency of abnormality on					
	Overall		Cognitive scale		Motor scale		Language scale	
Variable	n	%	n	%	n	%	n	%
**Gender**								
Male	45	42.5	16	35.6	14	31.1	18	40.0
Female	61	57.5	30	49.2	26	42.6	27	44.3
**Place of birth**								
Born before arrival	4	3.8	2	50	1	25.0	1	25.0
Inborn	98	93.3	44	44.9	38	38.8	43	43.9
Outborn at another clinic or hospital	2	2.9	0	0.0	0	0.0	0	0.0
**Mode of delivery**								
Normal delivery	34	32.1	16	47.1	12	35.3	14	41.2
Vaginal breech	2	1.9	0	0.0	0	0.0	0	0.0
Caesarean	68	64.8	28	41.2	26	38.2	30	44.1
**Presentation**								
Breech	11	10.5	4	36.4	1	9.1	3	27.3
Transverse	1	0.9	0	0	0	0.0	0	0.0
Vertex	93	88.6	41	44.1	7	7.5	41	44.1
**Hypothermia at birth**								
No	102	96.2	46	45.1	39	38.2	45	44.1
Yes	4	3.8	0	0.0	1	25.0	0	0.0
**Resuscitation at birth**								
No	70	66.0	30	42.9	21	30.0	25	35.7
Yes	36	34.0	16	44.4	19	52.8	20	55.6
**Sepsis (Early/late onset)**								
No	91	86.7	42	46.2	33	36.3	38	41.8
Yes	14	13.3	4	28.6	7	50.0	7	50.0
**Blood transfusion given**								
No	74	69.8	36	48.7	30	40.5	32	43.2
Yes	32	30.2	10	31.3	10	31.3	13	40.6
**KMC care done**								
No	10	9.4	4	40.0	4	40.0	6	60.0
Yes	95	89.6	41	43.2	35	36.8	38	40.0
**Ventilatory support given (IPPV/NCPAP)**								
No	82	77.4	35	42.7	29	35.4	32	39.0
Yes	24	22.6	11	45.8	11	45.8	13	54.2
**Surfactant given**								
No	91	85.8	39	42.9	33	36.3	36	39.6
Yes	15	14.2	7	46.7	7	46.7	9	60.0
**HIV exposed**								
No	48	67.6	23	47.9	21	43.8	20	41.7
Yes	23	32.4	10	43.5	8	34.8	10	43.4
**Antenatal steroids given**								
No	61	57.6	29	47.5	21	34.4	25	41.0
Yes	45	42.5	17	37.8	19	42.2	20	44.4
**Syphilis exposed**								
No	105	99.1	46	43.8	39	37.1	44	41.9
Yes	1	0.9	0	0.0	1	100.0	1	100.0
**PDA**								
No	101	95.3	45	44.6	38	37.6	42	41.6
Yes	5	4.7	1	20.0	2	40.0	3	60.0
**Hypotension**								
No	105	99.1	46	43.8	40	38.1	45	42.9
Yes	1	0.9	0	0.0	0	0.0	0	0.0
**NEC**								
No	104	98.1	45	43.3	39	37.5	45	43.3
Yes	2	1.9	1	50.0	1	50.0	0	0.0
**PVL**								
No	104	98.1	45	43.3	39	37.5	44	42.3
Yes	2	1.9	1	50.0	1	50.0	1	50.0
**Dilated ventricles**								
No	100	95.2	43	43.0	38	38.0	42	42.0
Yes	5	4.8	3	60.0	2	40.0	3	60.0
**IVH Grade**								
0	91	85.9	41	45.1	35	38.5	40	44.0
1	1	0.9	0	0.0	1	100.0	0	0.0
2	12	11.3	5	41.7	3	25.0	4	33.3
3	2	1.9	0	0.0	1	50.0	1	50.0
**Birth Weight**								
< 750 g	3	2.8	1	33.3	1	33.3	2	66.7
750-900 g	5	4.7	0	0.0	1	20.0	0	0.0
900-1000 g	8	7.6	4	50.0	5	62.5	4	50.0
1000-1250 g	53	50.0	24	45.3	23	43.4	25	47.2
≥ 1250 g	37	34.9	17	46.0	10	27.0	14	37.8
**Gestational Age**								
≤ 28	26	24.5	11	42.3	9	34.6	11	42.3
28-30	26	24.5	10	38.5	10	38.5	10	38.5
30-32	27	25.5	11	40.7	11	40.7	14	51.9
32-34	19	17.9	11	57.9	8	42.1	7	36.8
> 34	8	7.6	3	37.5	2	25.0	3	37.5
**Duration of Hospital stay **(days)								
< 30	27	25.5	12	44.4	6	22.2	7	25.9
30-40	30	28.3	14	46.7	12	40.0	19	63.3
40-50	21	19.8	8	38.1	10	47.6	6	28.6
≥ 50	28	26.4	12	42.9	12	42.9	13	46.4
**Duration of Intensive Care **(days)								
< 6	9	60.0	4	44.4	4	44.4	6	66.7
≥ 6	6	40.0	2	33.3	2	33.3	3	50.0
**Duration of supplemental oxygen **(days)								
< 10	70	73.7	31	44.3	24	34.3	26	37.1
10-30	11	11.6	3	27.3	4	36.4	5	45.5
≥ 30	14	14.7	6	42.9	7	50.0	9	64.3
**5 minute Apgar**								
< 6	18	18.0	7	38.9	10	55.6	7	38.9
≥ 6	82	82.0	37	45.1	29	35.4	37	45.1

Although most of the cross-tabulations in Table 3 had small numbers, we note that 11% (4), 14% (5) and 11% (4) of babies who required resuscitation at birth had abnormal cognitive, motor and language scores compared to those who did not (7.1%, 8.7% and 8.7% respectively). Although numbers are small, 1 out of 2 babies with PVL had abnormality on each subscale compared to only 10% among those without PVL.

### Univariate analysis - Logistic regression

All factors which were significantly associated with poor outcome at univariate level were considered in the multivariable logistic regression. Such factors included gender and blood transfusion for abnormal Bayley cognitive score; 5 minute Apgar score, resuscitation at birth, syphilis results and antepartum haemorrhage for abnormal motor score; and duration of hospital stay, duration on supplemental oxygen and resuscitation at birth for an abnormal language score. For example, at univariate level analysis, babies were more likely to be abnormal on the Bayley motor scale if they were resuscitated at birth (OR: 2.61, 95% CI: 1.14, 5.98) but less likely if their 5 minute Apgar score was more than 6 (OR: 0.44, 95% CI: 0.16, 1.23).

### Multivariable Analysis - Logistic regression

Although not significant and could be due to chance, the results of the multiple logistic regression show that female babies were 1.76 (95% CI: 0.79, 3.92) times more likely to have an abnormal Bayley cognitive score whereas babies who had blood transfusion were less likely (OR: 0.48, 95% CI: 0.20, 1.16) compared to those who had not although these adjusted results were not significant.

The adjusted analysis for the Bayley motor scale showed that resuscitation at birth was not significant (OR: 2.13, 95% CI: 0.85, 5.31). Logistic regression results for language scale showed that duration of hospital stay and resuscitation at birth (p = 0.039) were statistically significant factors. Those who stayed in hospital between 30 to 40 days were 7.41 times (OR: 95% CI: 1.88, 29.18) more likely to have an abnormal language score than those who stayed for less than 30 days. Those who were resuscitated at birth (OR: 2.90, 95% CI: 1.06, 7.95) and with a maternal parity of 4 (OR: 11.80, 95% CI: 1.01, 138.36) were at higher risk of an abnormal language score than those who were not resuscitated and those with a maternal parity of 1 respectively.

### Multivariable Analysis - Linear regression

Various multiple linear regression analyses which included only factors univariately associated with the outcome variables were performed. Hypothermia (p = 0.007) and PVL (p = 0.044) were significant risk factors for the cognitive score as outcome.

For the BSID 111 motor subscale, we found that PVL (p = 0.008) was a significant factor and that duration on oxygen (p = 0.064) was a borderline significant risk factor for BSID 111 motor score. Only PVL was statistically significantly associated with BSID 111 language score (p = 0.014).

## Discussion

The present study provides information on neurodevelopmental outcome in a cohort of VLBW infants in Johannesburg, South Africa. The mean BSID 111 scores at a median corrected age of 16.48 months age were within normal limits for the cognitive, motor and language subscales. Furthermore, considering 70 as the cutoff for the BSID 111 score, only 15.1% of infants had an abnormality on a single subscale, while 3.7% were affected in all 3 areas. However, the mean score on each subscale was less than the anticipated population mean of 100. Almost one third of patients were considered to be "at risk", with a BSID 111 score between 70 and 85, indicating that this is a high risk group of children likely to have long term developmental problems who warrant ongoing monitoring and intervention. Currently long term follow up of ex preterm infants in developing countries is frequently limited to those with obvious handicap due to insufficient resources.

This group of VLBW infants represents a select group, as ventilatory support was not offered to infants with a birth weight less than 900 grams, resulting in a very low survival of infants of lower birth weight. Although other neonatal units in developing countries may not apply strict birth weight cut offs for ventilation, or the birth weight cut offs may be different, the challenges of being unable to ventilate all preterm infants who require support will be very similar.

The neurodevelopmental outcome results in the present study are similar to those of Cooper and Sandler [15] at Chris Hani Baragwanath hospital, Soweto, in the 1990s. They found 15.3% of VLBW infants had an abnormal BSID score at follow up. The results are also comparable with those reported in the literature. In a follow up study reported from Bangladesh [14], only 32% of VLBW infants were reported as neurodevelopmentally intact at 12 months of age. Many follow up studies are confined to extremely premature infants, born between 22 and 26 weeks gestation. Up to one quarter of these infants will have at least one major disability in childhood [21]. The rates of disability in VLBW infants or those born after 26 weeks gestation are heterogeneous. Of a group of babies born < 29 weeks gestation between 1985 and 1987, only 31% had no physical or educational handicap and 21% had at least one severe disability at 7 years of age [9]. A group of infants born at a median age of 28 weeks and assessed at a median corrected age of 18 months showed normal outcome in 59%, borderline function in 26% and abnormal outcome in 15% [22] The survival without neurodevelopmental disability of infants born < 30 weeks gestation improved from 62% in infants born between 1985 and 1986 to 81% for those born between 2005 and 2006 [23]. In the EPIPAGE study [3] in France, infants born below 32 weeks gestation between 1997 and 2001 showed normal profiles at 6 to 10 years of age in 68%, minor disorders in 18% and major disorders in 14%. A group of Finnish infants born between 2001 and 2006 were assessed at 2 years of age and 9.9% were found to have neurodevelopmental impairment. The outcome of preterm infants below 32 weeks born at a tertiary centre in Ankara in Turkey, 16.6% were found to have minor neurological dysfunction and 8.3% to have cerebral palsy at a median age of 25.85 months [24]. Of this group, 24.8% had a low Bayley Psychomotor development index and 25.4% a low Bayley Mental Development index.

In the present study, cystic PVL was associated with poor cognitive, motor and language function. Further, duration of supplemental oxygen, prolonged hospitalisation, resuscitation at birth and increased maternal parity were associated with poor outcome. Duration of intensive care showed a trend towards worse outcome. These findings were similar to those reported in other studies where NEC [24-28], gender [7,26,29-33], chronic lung disease [3,7,32,34], respiratory distress [35,36], multiple birth [7], HIV infection [37], cranial sonar findings [29,38-41], particularly PVL [15,26,31,42,43] and intraventricular haemorrhage, [26,44,45]), neonatal seizures [26,44], perinatal asphyxia [41,44,45], neonatal sepsis [27,41], postnatal steroids [31,33,34] and the duration of assisted ventilation [24,26,30] have all been associated with adverse neurodevelopmental outcome in VLBW infants. Gestational age [3,35,46] and birth weight [10,30,46] have both been reported as predictors of poor neurodevelopmental outcome. This was not the case in the present study, which almost certainly reflects the ventilation policy in the unit at the time, where babies with a birth weight below 900 grams were not offered ventilation, so survival in this birthweight category is low [47]. Other neonatal factors such as treated hypotension [48] were not associated with poor outcome in the current study - possibly due to small numbers of affected patients. HIV exposure, ethnicity and KMC were not predictive of outcome in the present study. The present study shows no difference between males and females with regard to neurodevelopmental outcome, which is contrary to findings in other studies where male gender is associated with worse outcome [29,30]. The reason for this is unclear and would have to be confirmed in future research.

### Limitations of the study

The rate of cerebral palsy (CP) is of great importance when considering the outcome of preterm infants. The reported number of CP cases in the present study is low (3.7%). This could be an underestimation of the true rate of cerebral palsy in this population as the age of assessment in the present study is too low to reliably report on the rate of cerebral palsy. The diagnosis of cerebral palsy in many children can only be made reliably after the age of 2 years [49]. A significant number of preterm infants followed up in one study had a change in neurological diagnosis made at 18 months as compared to 30 months and it therefore may be necessary to delay the diagnosis of cerebral palsy in some children [50]. It is likely that those infants, who present later, will be relatively mild in comparison to those who present early. It is also possible that some cases of CP were among those lost to follow up. However, the CP rate in this population would be expected to be low, as the sickest and smallest of these infants do not survive [47]

Although the rate of follow up achieved of 74.6% is acceptable and comparable to other reports, it is possible that a number of handicapped children were lost this way. The most common reason for non compliance was relocation of the parents to their place of origin, some as far afield as Malawi and Tanzania. Parents also return to work and find it difficult to bring their children for follow up after the first few visits. Transport and hospital strikes also resulted in the loss to follow up of some patients. Five patients returned to follow up on the incorrect day when a physiotherapist was unavailable, so did not have a Bayley assessment; these children were all clinically normal.

The BSID 111 has been tested on black South African children, between 0 and 18 months of age, who did not have risk factors or pre-existing conditions. The results showed that these children performed well and were often above average (a composite score > 100 on each BSID 111 subscale), confirming that the BSID 111 is suitable for use in line with previous studies in this population [51,52].

Another limitation of the study is that children had assessments done at different ages for the reasons outlined above. Ideally all assessments should have been done at the same age on all patients. As previously noted, the age of assessment influences the result obtained in the BSID scores. For this reason and high missing data on follow-up visits, we reported the latest BSID 111 in each patient as the outcome variable and did not conduct a repeated measures analysis, which would have been ideal.

## Conclusions

This study provides neurodevelopmental outcome data in a group of VLBW infants in Johannesburg, South Africa. The prevalence of cerebral palsy and severe handicap is low and is similar to that reported from other developing countries. However, this low rate of handicap may reflect the low survival rate of infants with a birth weight below 900 grams. Also, the mean scores on each of the BSID 111 subscales although within normal limits, were relatively low and one third of the patients were identified as being at risk of developmental problems, with a BSID 111 score of between 70 and 85. Thus, VLBW infants in this setting are a high risk group of patients likely to have learning and other difficulties at school going age and warrant long-term follow up.

## List of abbreviations

The following abbreviations are found in the article: BSID 111: Bayley Scales of Infant and Toddler Development (Version 111); CMJAH: Charlotte Maxeke Johannesburg Academic Hospital; CP- cerebral palsy; ELBW: Extremely low birth weight (≤ 1000 grams); IPPV: intermittent positive pressure ventilation; KMC: kangaroo mother care; NCPAP: nasal continuous positive airways pressure; NEC: Necrotising enterocolitis; PDA: patent ductus arteriosus; PVL: Cystic periventricular leukomalacia; VLBW: Very low birth weight (≤ 1500 grams)

## Competing interests

The authors declare that they have no competing interests.

## Authors' contributions

DEB was the main researcher, conceptualized and conducted the follow up study, collated the data and wrote up the manuscript. JP advised on the study design, conducted the BSID 111 on the patients and reviewed the manuscript. TC conducted the statistical analysis and reviewed the manuscript. NH conducted the BSID 111 and reviewed the manuscript. PC assisted with the study design, follow up of patients and review of manuscript. The final submission of the manuscript was approved by all authors.

## Pre-publication history

The pre-publication history for this paper can be accessed here:

http://www.biomedcentral.com/1471-2431/12/11/prepub
